# Use of an Electronic Health Record Algorithm to Identify Persons With Human Immunodeficiency Virus Out Of Care in United States Emergency Departments

**DOI:** 10.1016/j.acepjo.2026.100513

**Published:** 2026-09-30

**Authors:** Douglas A.E. White, Jacob Manteuffel, Molly Burns, Montana Jewett, Cedric Rodriguez, Gabriela Regalado, Cinthya Mujica Pinto, Kevin Del Angel, Christian Loszewski, Dalton Bender, Jo-Ann Rammal, Shannon Payne, Sharon Zahul, William Grace, Indira Brar, Joseph Miller, Mit Patel, Erik Anderson, Bijou Hunt

**Affiliations:** 1 Department of Emergency Medicine, Alameda Health System, Oakland, California, USA; 2 Division of Public Health, Department of Emergency Medicine, Henry Ford Hospital, Detroit, Michigan, USA; 3 Division of Infectious Diseases, Department of Medicine, Henry Ford Hospital, Detroit, Michigan, USA; 4 Division of Infectious Diseases, Department of Medicine, Sinai Infectious Disease Center, Sinai Chicago, Chicago, Illinois, USA

**Keywords:** HIV care continuum, persons with diagnosed HIV, out-of-care, electronic health records, emergency department, linkage to care

## Abstract

**Objective:**

Emergency department (ED) human immunodeficiency virus (HIV) screening programs are designed primarily to identify persons with undiagnosed HIV and, therefore, systematically exclude persons with diagnosed HIV (PWHDx). PWHDx who are out of care (OOC) contribute disproportionately to HIV transmission and are a priority population within Ending the HIV Epidemic (EHE) initiatives. Because PWHDx OOC are not routinely identified through screening, complementary strategies are needed to support re-engagement in care. We evaluated an electronic health record algorithm (EHRa) designed to identify PWHDx presenting to the ED. We then determined HIV care status, characterized differences between patients in care (IC) and OOC, and quantified the incremental yield of EHRa-based identification beyond routine HIV screening.

**Methods:**

We conducted a retrospective, cross-sectional study across 3 EDs with universal HIV screening. Adults (≥18 years) presenting between February and July 2024 who screened HIV positive or were identified through the EHRa were included. Data from HIV screening databases and EHRa reports were analyzed using descriptive statistics and logistic regression.

**Results:**

Among 88,501 adult ED visits, 798 persons with HIV were identified (0.9%): 5.1% newly diagnosed, 21.6% PWHDx OOC, and 73.3% PWHDx IC. The EHRa identified 589 PWHDx who were not captured through routine screening, including 113 (19.2%) who were OOC. Compared with IC patients, PWHDx OOC had higher AIDS prevalence (37.5% vs 18.6%) and lower viral suppression (17.6% vs 83.3%). OOC status was independently associated with cisgender female gender, housing instability, limited phone access, and being uninsured.

**Conclusions:**

A simple, locally generated EHRa substantially increased identification of PWHDx in the ED, including many OOC patients who were not captured through routine screening. A simple, locally generated EHRa demonstrated substantial incremental yield for identifying PWHDx in the ED, including many OOC patients who were not captured through routine screening. EHR-based identification strategies may help identify and re-engage PWHDx OOC and support national EHE goals.

**The Bottom Line**

Emergency departments (EDs) with human immunodeficiency virus (HIV) screening programs are effective at identifying people with undiagnosed HIV. Although some people with diagnosed HIV are identified through repeat testing, these programs are designed to exclude them and therefore do not systematically identify those who have fallen out of care.

In this retrospective study of 88,501 adult ED visits at 3 hospitals with HIV screening programs, an electronic health record algorithm (EHRa) identified 798 people with previously diagnosed HIV. Of these, 172 (21.6%) were out of care, and 113 (65.7%) were identified through the EHRa without repeat HIV testing. These findings suggest that EHR-based identification may help address an important gap in the HIV care continuum by supporting re-engagement in care.

## 1 Introduction

### 1.1 Background

In 2019, the United States (U.S.) Department of Health and Human Services launched the Ending the HIV Epidemic (EHE) initiative to reduce new HIV infections by 90% by 2030.^1^ The EHE emphasizes 2 central strategies: (1) routine HIV screening to identify individuals unaware of their HIV status and (2) re-engagement in care for persons with diagnosed HIV (PWHDx) who are out of care (OOC).^2^ Both strategies are essential to reducing viral transmission and achieving national prevention goals.

Over the past 2 decades, emergency departments (ED) have become integral to national EHE efforts by implementing routine opt-out HIV screening programs that identify patients with previously undiagnosed infection.^3^, ^4^, ^5^, ^6^ Although these programs have effectively advanced the “diagnosis” pillar of EHE, the “re-engagement” pillar has received less attention, partly because the OOC population in ED settings has been, until recently, underrecognized.^7^

Recent Centers for Disease Control and Prevention (CDC) estimates indicate that nearly half of the more than one million PWHDx in the U.S. are OOC.^8^ Many are not receiving antiretroviral therapy (ART) and remain virally unsuppressed, contributing disproportionately to ongoing HIV transmission.^9^^,^^10^ These findings highlight the urgent need for interventions to address this care gap.

ED HIV screening programs are designed primarily to identify undiagnosed infection and, therefore, generally exclude patients with known HIV diagnoses. As a result, PWHDx—including those disengaged from care—are not routinely identified through screening workflows. Nevertheless, repeat-positive HIV tests are common in ED screening programs and often identify previously diagnosed individuals, many of whom are OOC.^11^, ^12^, ^13^, ^14^ In many EDs, repeat-positive results consistently outnumber new diagnoses, underscoring the ED as an important point of contact for PWHDx disconnected from care and highlighting the limitations of screening alone as a strategy for identifying this population.^11^, ^12^, ^13^, ^14^ A complementary approach is, therefore, needed to systematically identify and re-engage PWHDx OOC.

### 1.2 Importance

These findings highlight the need for structured ED workflows to identify and re-engage PWHDx OOC. Published reports describing such systematic strategies remain limited,^15^, ^16^, ^17^ underscoring an important opportunity for innovation.^7^ Developing reliable approaches to identify PWHDx OOC, quantify their prevalence among ED patients, and characterize barriers to re-engagement, are essential steps toward improving linkage. Such insights can guide ED workflows and institutional policies that align operations with EHE objectives and strengthen the HIV care continuum.

### 1.3 Goals of This Investigation

We conducted this study to evaluate the performance of an electronic health record algorithm (EHRa) designed to identify PWHDx presenting to the ED. We sought to determine the number and proportion of PWHDx OOC and to identify potential barriers to re-engagement. We also compared these outcomes with results from existing ED HIV screening programs to illustrate the incremental yield of the EHRa as a complementary identification strategy for PWHDx OOC who are excluded from routine screening by design.

## 2 Methods

### 2.1 Study Design and Setting

This was a retrospective, cross-sectional cohort study conducted across 3 U.S. EDs within 2 health care systems: Alameda Health System–Highland Hospital (AHS-HH) in Oakland, California (annual ED census ≈57,000 visits), Alameda Health System–San Leandro Hospital (AHS-SLH) in San Leandro, California (≈35,000 visits), and Henry Ford Hospital (HFH) in Detroit, Michigan (≈92,000 visits). AHS-HH and HFH are academic, regional trauma centers with accredited emergency medicine residency programs; AHS-SLH is a community ED.

This report adheres to the Strengthening the Reporting of Observational Studies in Epidemiology (STROBE) statement.^18^ The study was approved by the HFH Institutional Review Board with a waiver of written informed consent.

### 2.2 Interventions

#### 2.2.1 HIV screening program

All participating sites maintain opt-out, universal HIV screening and physician-initiated diagnostic testing consistent with CDC recommendations.^19^ HIV screening is implemented through an EHR-embedded algorithm within the Epic laboratory ordering workflow (Epic Systems Corporation, Verona, WI), which automatically orders an HIV screening test for eligible patients undergoing routine bloodwork. Patients are excluded from HIV screening if they are <18 years old, have undergone HIV testing within the prior 12 months, or are identified as PWHDx based on a documented HIV diagnosis in their medical history or a prior positive HIV laboratory result recorded in the EHR. These 2 exclusion criteria are distinct: the 12-month testing window applies to patients without a known HIV diagnosis who have been recently tested, whereas the diagnosis-based exclusion applies to all patients with documented HIV-positive status, regardless of when they were last tested. This design intentionally focuses screening resources on potentially undiagnosed patients and is not intended to capture patients with known HIV who may be OOC. Dedicated HIV navigators assist with counseling, results disclosure, and linkage to care for all patients testing positive. Screening programs were supported by Gilead Sciences’ FOCUS Program.^20^

#### 2.2.2 Electronic health record algorithm (EHRa)

Each health system developed an EHRa to identify ED PWHDx. The algorithm generated Microsoft Excel reports (Microsoft Corporation, Redmond, WA) using historical laboratory results and keyword searches of PMH fields retrievable within the Epic EHR (Table 1). All EHRa-identified patients were reviewed retrospectively—by infectious disease clinicians at HFH and by HIV navigators at AHS sites—and classified as in care (IC) or OOC according to site-specific procedures (Table 1). HFH defined patients as IC if they had documentation of an HIV clinic visit or HIV-related laboratory test within the prior year. At AHS sites, care status was determined by HIV navigators using a structured chart review process that incorporated laboratory results, antiretroviral prescription history, outpatient HIV appointment attendance, clinician documentation, and, when available, direct patient contact. Patients with evidence of ongoing HIV treatment or engagement in HIV medical care were classified as IC. Patients lacking evidence of active HIV care engagement after review of available information were classified as OOC. Unlike HFH, AHS did not apply a fixed time-based criterion for care engagement.

**Table 1:** Criteria used by sites to identify ED patients as previously diagnosed with HIV infection and to classify care status.

	Alameda Health System (Highland and San Leandro Hospitals)	Henry Ford Hospital
Electronic Health Record Algorithm for HIV identification	Previous diagnosis of HIV documented in the past medical history or problem list and/or a prior reactive HIV-1 antibody test or HIV RNA >20 copies/mL.	Previous diagnosis of HIV documented in the past medical history or problem list and/or a prior reactive HIV-1 antibody test.
Criteria for care status determination	HIV navigators determined care status (IC vs OOC) using clinical judgment after review of laboratory results, pharmacy records, progress notes, appointment histories, and patient interviews. Records were reviewed in local EHR and Care Everywhere systems (Epic Systems Corporation, Verona, WI). Patients not clearly in care were classified as OOC.	Infectious disease staff classified patients as IC if they had an HIV clinic visit or HIV-related laboratory test within the prior 12 months. Those not meeting these criteria were classified as OOC. Records were reviewed in local EHR and Care Everywhere systems (Epic Systems Corporation, Verona, WI) and verified against Michigan Department of Health and Human Services data.

ED, emergency department; EHR, electronic health record; RNA, ribonucleic acid; IC, in care; OOC, out of care.

EHRa reports were generated and reviewed retrospectively—at staff-determined intervals and after patients’ ED visits. The algorithm was not integrated into real-time linkage workflows; its sole purpose during this pilot was to identify the study cohort and evaluate feasibility and yield.

### 2.3 Selection of Participants

All adults (aged ≥18 years) presenting to a study ED between February 1 and July 31, 2024 were eligible. Patients were included if they (1) tested HIV positive during ED screening or (2) were identified by the EHRa as a PWHDx. Patients with false-positive HIV test results and those identified by the EHRa who were later determined not to be PWHDx were excluded.

### 2.4 Measurements

Data were abstracted from multiple sources and manually entered into a data collection instrument in REDCap for all patients identified by the EHRa and/or for those who completed HIV screening.^21^^,^^22^ Data were derived from HIV program administrative databases used for quality improvement and grant reporting, EHRa reports, chart abstraction, laboratory reports, and patient self-report. The EHRa reports included all PWHDx identified up to the report generation date, capturing patients identified through both historical data and contemporaneous ED screening during the reporting window. For patients with multiple visits, only data from the first encounter were analyzed.

Data included demographics (age, race, ethnicity, gender), HIV test results (antigen/antibody assays, antibody differentiation, HIV RNA, CD4), care status (new diagnosis [ND], IC, OOC), and selected social determinants of health (housing, language, phone access, insurance, and substance use). PWHDx were classified as identified by the EHRa if they appeared in the EHRa report and were confirmed HIV positive. Patients identified by the EHRa without an ED HIV test during the index visit were classified as EHRa-only identifications, whereas those with a positive ED test were classified as screening identifications.

For all PWHDx, CD4 count and viral load results obtained within ±60 days of the first visit were analyzed. AIDS was defined as CD4 <200 cells/μL; viral suppression as HIV RNA <200 copies/mL; and undetectable viral load as <20 copies/mL.

Each site collected and managed data independently. Deidentified data were aggregated at a single site for analysis.

### 2.5 Outcomes

The primary outcome was the total number of ED PWHDx identified, including the number and proportion classified as OOC; the secondary outcome was the number of additional OOC PWHDx identified through the EHRa who were not detected by screening, quantifying the incremental yield of algorithm-based identification. A tertiary analysis compared characteristics between OOC and IC patients to identify potential barriers to care.

### 2.6 Data Analysis

Analyses were conducted on deidentified data using Stata version 14 (StataCorp, College Station, TX). The unit of analysis was the individual patient. Descriptive statistics summarized continuous variables as medians (IQR) or means (SD) and categorical variables as proportions with 95% confidence intervals (CI). Bivariate comparisons used the Wilcoxon rank-sum, chi-square, or Fisher exact tests, as appropriate. Logistic regression models were constructed to identify factors associated with OOC status compared with IC status; the model was developed using expert consensus on demographics as well as clinical variables that may impact engagement in HIV care. Encounters with missing data were not included in the regression model.

## 3 Results

During the 6-month study period, there were 88,501 ED visits among adults aged ≥18 years. Fig summarizes outcomes of the EHRa and HIV screening program. The EHRa identified 771 patients, of whom 14 did not have HIV and 168 underwent repeat HIV screening, leaving 589 PWHDx, who would not have been identified by screening alone. Among these, 476 (80.8%) were IC and 113 (19.2%) were OOC. During the same period, 17,710 HIV screening tests were completed, identifying 209 PWH (1.2% positivity rate). Of these, 41 (19.6%) were newly diagnosed (ND), and 168 (80.4%) were PWHDx. Among the rescreened PWHDx, 109 (64.9%) were IC and 59 (35.1%) were OOC.

**Figure:**
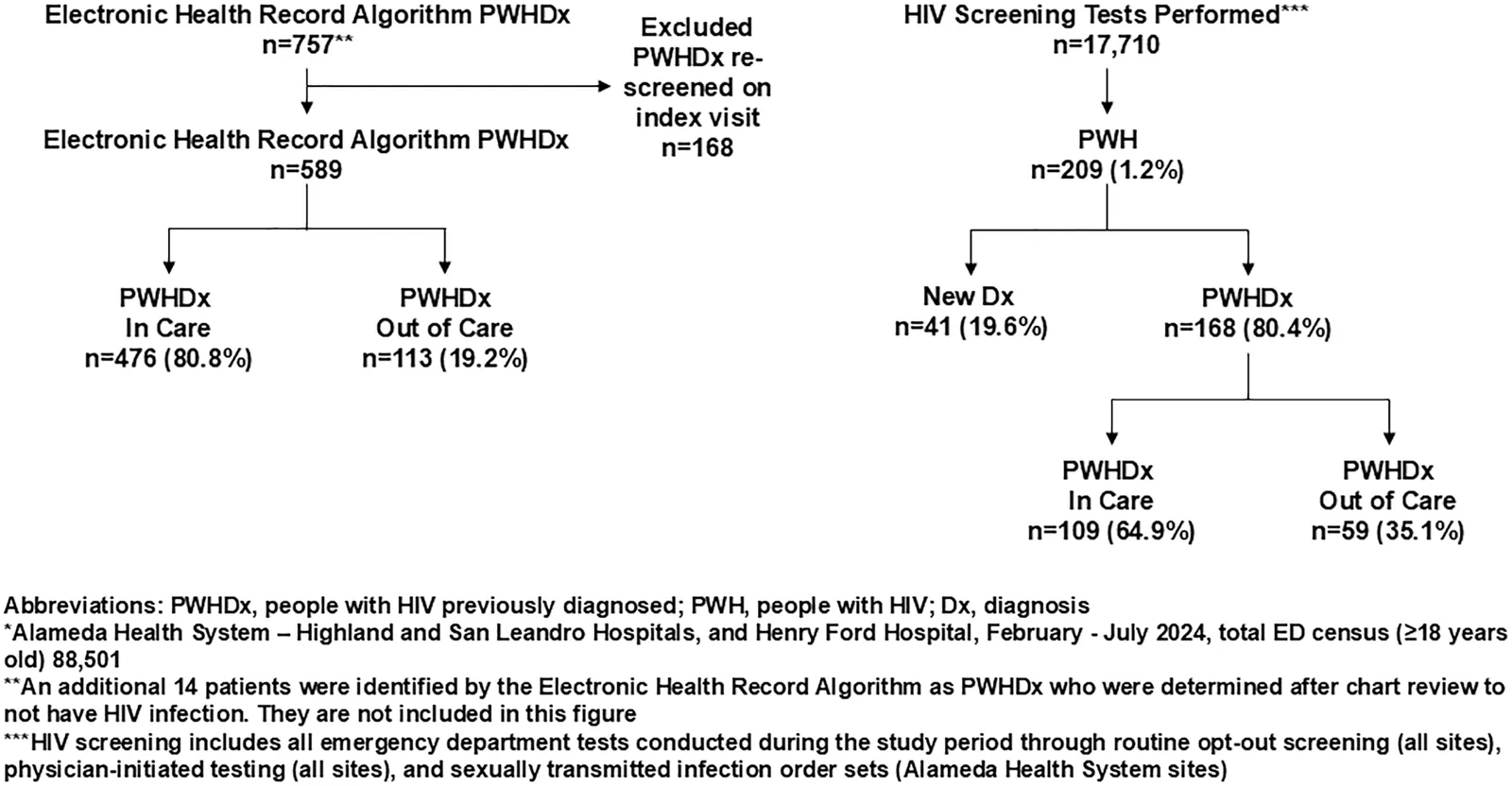
Flow diagram of electronic health record algorithm and HIV screening results among emergency department patients with HIV infection.∗

Across all sites, the overall prevalence of HIV among ED patients was 0.9% (Table 2). The majority were PWHDx IC (73.3%), followed by PWHDx OOC (21.6%), and ND (5.1%). Most were cisgender men (69%) and Black or Latinx (85%). Site-specific patient characteristics appeared broadly similar across hospitals.

**Table 2:** Characteristics of emergency department patients with HIV infection, stratified by site.

	All Sites (n = 798)	AHS-HH (n = 261)	AHS-SLH (n = 119)	HFH (n = 418)
Census ≥18 years, n (% HIV+)	88,501 (0.9)	29,126 (0.9)	16,273 (0.7)	43,102 (1.0)
HIV diagnosis, n (%)				
New	41 (5.1)	15 (5.8)	6 (5.0)	20 (4.8)
PWHDx out of care	172 (21.6)	60 (23.0)	20 (16.8)	92 (22.0)
PWHDx in care	585 (73.3)	186 (71.3)	93 (78.2)	306 (73.2)
Age mean (SD) years^a^	47.6 (14.7)	48.3 (15.4)	48.8 (13.4)	46.4 (14.8)
Gender identity, n (%)				
Cisgender male	550 (68.9)	192 (73.6)	68 (57.1)	290 (69.4)
Cisgender female	202 (25.3)	56 (21.5)	43 (36.1)	103 (24.6)
Transgender male	4 (0.5)	1 (0.4)	0	3 (0.7)
Transgender female	37 (4.6)	11 (4.2)	6 (5.0)	20 (4.8)
Gender nonconforming/nonbinary/other	5 (0.6)	1 (0.4)	2 (1.7)	2 (0.5)
Race/ethnicity, n (%)				
Hispanic	108 (13.5)	65 (24.9)	26 (21.9)	17 (4.1)
Black	574 (71.9)	137 (52.5)	73 (61.3)	364 (87.1)
White	80 (10.0)	39 (14.9)	14 (11.8)	27 (6.5)
Biracial/multiracial	5 (0.6)	3 (1.1)	1 (0.8)	1 (0.2)
Asian	13 (1.6)	11 (4.2)	1 (0.8)	1 (0.2)
Other^b^	16 (2.0)	4 (1.5)	4 (3.3)	8 (1.9)
Language, n (%)				
English	725 (90.9)	210 (80.5)	109 (91.6)	406 (97.1)
Spanish	56 (7.0)	38 (14.5)	9 (7.6)	9 (2.1)
Other	17 (2.1)	13 (5.0)	1 (0.8)	3 (0.7)
Disposition, n (%)				
Admit	201 (25.2)	55 (21.1)	20 (16.8)	126 (30.1)
Discharge	597 (74.8)	206 (78.9)	99 (83.2)	292 (69.9)

AHS-HH, Alameda Health System–Highland Hospital; AHS-SLH, Alameda Health System–San Leandro Hospital; HFH, Henry Ford Hospital; PWHDx, people with HIV infection previously diagnosed; SD, standard deviation; n, number.

Totals may not sum to 100% because of missing Race/Ethnicity data for 2 patients.

a Age mean (SD) computed as a weighted average across sites.

b Other includes Alaska Native, American Indian, Native Hawaiian, Pacific Islander.

Demographic characteristics of PWH by diagnosis and care status are presented in Table 3. Although distributions were broadly similar, ND patients tended to be younger, whereas PWHDx were older. A descriptively higher proportion of PWHDx OOC were transgender women, though this subgroup was small and this observation should be interpreted with caution. No substantial differences in race, ethnicity, or language distributions were observed between PWHDx OOC and IC.

**Table 3:** Characteristics of emergency department patients with HIV infection, stratified by new diagnosis, previously diagnosed out of care, and previously diagnosed in care.

Characteristic	New HIV diagnosis (n = 41)	Previously diagnosed out of care (n = 172)	Previously diagnosed in care (n = 585)
Age, mean (SD), years	32.8 (10.3)	43.6 (12.6)	49.5 (14.9)
Gender identity, n (%)			
Cisgender male	28 (68.3)	107 (62.2)	415 (70.9)
Cisgender female	12 (29.3)	50 (29.1)	140 (23.9)
Transgender male	0 (0.0)	1 (0.6)	3 (0.5)
Transgender female	1 (2.4)	12 (7.0)	24 (4.1)
Gender nonconforming/nonbinary/other	0 (0.0)	2 (1.2)	3 (0.5)
Race/ethnicity, n (%)			
Hispanic	9 (22.0)	23 (13.4)	76 (13.0)
Black / African American	24 (58.5)	125 (72.7)	425 (72.6)
White	5 (12.2)	16 (9.3)	59 (10.0)
Biracial/multiracial	0 (0.0)	3 (1.7)	2 (0.3)
Asian	1 (2.4)	2 (1.2)	10 (1.7)
Other^a^	2 (4.9)	2 (1.2)	12 (2.1)
Primary language, n (%)			
English	36 (87.8)	157 (91.3)	532 (90.9)
Spanish	4 (9.8)	12 (7.0)	40 (6.8)
Other	1 (2.4)	3 (1.7)	13 (2.2)
ED disposition, n (%)			
Admit	12 (29.3)	38 (22.1)	151 (25.8)
Discharge	29 (70.7)	134 (77.9)	434 (74.2)

PWHDx, people with HIV infection previously diagnosed; SD, standard deviation; N, number.

Totals may not sum to 100% because of missing Race/Ethnicity data for 2 patients.

a Other includes Alaska Native, American Indian, Native Hawaiian, and Pacific Islander.

Table 4 summarizes CD4 counts and HIV RNA viral loads at the index visit. Among patients with available CD4 data, AIDS was observed more frequently among PWHDx OOC (37.5%) than among ND (15.8%) or PWHDx IC (18.6%). Among those with viral load testing, PWHDx OOC had lower rates of viral suppression (27.9%) and undetectable viral load (17.6%) than PWHDx IC (83.3% and 68.5%, respectively).

**Table 4:** CD4 and HIV viral load results of emergency department patients with HIV infection, stratified by new diagnosis, previously diagnosed out of care, and previously diagnosed in care.

Characteristic	New HIV diagnosis (n = 41)	Previously diagnosed out of care (n = 172)	Previously diagnosed in care (n = 585)
CD4 measured within ±60 days, n (%)	19 (46.3)	56 (32.6)	226 (38.6)
CD4 count, cells/μL, median (IQR)	354 (259–569)	333 (123–519)	489 (267–803)
CD4 count <200 cells/μL, n (%)	3 (15.8)	21 (37.5)	42 (18.6)
HIV RNA viral load measured within ±60 days, n (%)	24 (58.5)	68 (39.5)	251 (42.9)
HIV RNA viral load, copies/mL, median (IQR)	144,824 (16,000–1,233,000)	18,650 (140–171,000)	0 (0–39)
Viral suppression (<200 copies/mL), n (%)	0 (0)	19 (27.9)	209 (83.3)
Undetectable viral load (<20 copies/mL), n (%)	0 (0)	12 (17.6)	172 (68.5)

PWHDx, people with HIV infection previously diagnosed; IQR, interquartile range; RNA, ribonucleic acid; n, number.

Patterns of social and behavioral factors are shown in Table 5. Several indicators of social vulnerability were more common among PWHDx OOC than among PWHDx IC. OOC patients more frequently reported housing instability (49.4% vs 24.3%), substance use disorders (54.1% vs 40.3%), lack of phone access (15.1% vs 1.9%), and absence of health insurance (6.4% vs 2.4%).

**Table 5:** Barriers to care and substance use of emergency department patients with HIV infection, stratified by whether new, previous diagnosis in care, or previous diagnosis out of care, Alameda Health System and Henry Ford Hospital.

	New HIV diagnosis (n = 41)	PWHDx out of care (n = 172)	PWHDx in care (n = 585)
Barriers to care, n (%)			
Unhoused/unstably housed	10 (24.4)	85 (49.4)	142 (24.3)
Incarcerated/in police custody	2 (4.9)	8 (4.6)	6 (1.0)
Mental health disorder	12 (29.3)	75 (43.6)	237 (40.5)
Substance use disorder	15 (36.6)	93 (54.1)	236 (40.3)
No phone	7 (17.1)	26 (15.1)	11 (1.9)
Uninsured	6 (14.6)	11 (6.4)	14 (2.4)
Substance use, n (%)			
Injection drug use	1 (2.4)	22 (12.8)	57 (9.7)
Alcohol	4 (9.8)	41 (23.8)	101 (17.3)
Methamphetamine	9 (22.0)	40 (23.3)	103 (17.6)
Opioids	3 (7.3)	28 (16.3)	63 (10.8)
Cocaine	5 (12.2)	39 (22.7)	97 (16.6)
Benzodiazepines	1 (2.4)	2 (1.2)	7 (1.2)
Ecstasy	2 (4.9)	2 (1.2)	11 (1.9)
Other	1 (2.4)	2 (1.2)	3 (0.5)

PWHDx, people with HIV infection previously diagnosed; SD, standard deviation; n, number.

Totals exceed 100% because patients could have ≥ 1 barrier to care or substance use disorders.

Multivariable logistic regression results are presented in Table 6. Compared with PWHDx IC, those OOC were more likely to be cisgender female (adjusted odds ratio [aOR] 1.7, 95% CI 1.1–2.5), unhoused (aOR 2.3, 95% CI 1.5–3.6), without a phone (aOR 7.2, 95% CI 3.3–16.1), or uninsured (aOR 2.8, 95% CI 1.2–6.7).

**Table 6:** Multivariable logistic regression of factors associated with being out of care among emergency department patients with HIV infection.

Variable	Odds ratio (95% CI)	Adjusted odds ratio (95% CI)
Emergency department		
AHS-HH	Ref	Ref
AHS-SLH	0.6 (0.4–1.2)	0.6 (0.3-1.1)
HFH	0.9 (0.6–1.4)	1.1 (0.7–1.8)
Age, years	1.0 (1.0–1.0)	1.0 (1.0–1.0)
Gender		
Male	Ref	Ref
Female	1.3 (0.9–2.00)	1.7 (1.1–2.5)
Transgender female	1.9 (0.9–4.0)	1.7 (0.8–3.7)
Other^a^	1.9 (0.5–7.9)	1.6 (0.4–7.1)
Race/ethnicity		
White	Ref	Ref
Black	1.1 (0.6–2.0)	1.3 (0.7–2.5)
Hispanic	1.1 (0.5–2.3)	0.9 (0.3–2.5)
Other^b^	1.2 (0.5–3.1)	1.2 (0.4-3.4)
Language		
English	Ref	Ref
Spanish	1.0 (0.5–2.0)	1.5 (0.5–4.1)
Other	0.8 (0.2–2.8)	0.9 (0.2–3.4)
Substance use		
No	Ref	Ref
Yes	1.7 (1.2–2.5)	1.5 (1.0–2.2)
Unhoused/unstably housed		
No	Ref	Ref
Yes	3.0 (2.1–4.3)	2.3 (1.5–3.6)
Mental health disorder		
No	Ref	Ref
Yes	1.1 (0.8–1.6)	0.8 (0.5–1.1)
No phone		
No	Ref	Ref
Yes	9.3 (4.5–19.2)	7.2 (3.3–16.1)
Uninsured		
No	Ref	Ref
Yes	2.8 (1.2–6.3)	2.8 (1.2–6.7)

AHS, Alameda Health System; HH, Highland Hospital; SLH, San Leandro Hospital; HFH, Henry Ford Hospital; CI, confidence interval; Ref, reference category.

There were 41 (5%) of patients not included in the model due to missing data.

a Transgender male, nonbinary, and other gender categories were excluded from the multivariable model because of small sample size.

b Other race/ethnicity includes Asian, Native Hawaiian or Pacific Islander, American Indian, and Alaska Native.

## 4 Limitations

This study has several limitations. All participating sites were EDs located within EHE priority jurisdictions,^23^ 2 of which were large academic hospitals (AHS-HH and HFH). The proportion of OOC patients may differ in nonacademic or non-EHE-priority settings,^24^ but inclusion of a smaller community site (AHS-SLH) enhances applicability.

Definitions and methods for classifying PWHDx as IC or OOC differed between institutions. Given these methodological differences, misclassification of care status is possible.^25^ However, the proportions of IC and OOC patients were highly consistent across sites, suggesting both approaches captured the underlying distribution with reasonable accuracy. Nevertheless, differential misclassification by site is possible and could have influenced OOC prevalence estimates and group comparisons.

The EHRa may not have identified all PWHDx, as it depended on data retrievable from Epic systems. Patients new to the health system or without updated records may have been missed. Additionally, patients receiving HIV care exclusively at external facilities may have been misclassified as OOC if documentation of that care was absent from the health system’s EHR. This limitation directly affects the primary outcome and could have biased estimates of OOC prevalence as well as the demographic and clinical comparisons between OOC and IC groups. The magnitude of this potential misclassification is unknown but may be substantial in settings where patients access care across multiple health systems. Future research should validate EHRa findings against navigator interviews and department of health surveillance records, and evaluate real time, integrated models to assess accuracy and linkage outcomes.

Because patients were clustered within 3 hospitals and care-status classification procedures differed somewhat across sites, we did not account for potential site-level correlation in the regression analyses. This may have resulted in underestimated standard errors and overstatement of statistical precision.

Our dataset classified all HIV tests as “screening,” which prevented us from distinguishing routine screening from clinician-initiated testing. In clinical practice, emergency physicians may order HIV tests in PWHDx to document HIV status, confirm patient self-report, or support re-engagement in care. Consequently, many repeat tests among PWHDx were likely misclassified as screening, leading to an overestimation of duplicate tests attributed to the screening program. Future studies should incorporate structured order fields or targeted chart review to more accurately characterize test origin.

## 5 Discussion

Many PWHDx in the U.S. remain disengaged from long-term HIV care, are not consistently prescribed ART, and fail to achieve viral suppression.^8^ These gaps in the HIV care continuum drive ongoing transmission and increase morbidity and mortality among PWH. Although many PWHDx do not access routine outpatient HIV care, they frequently receive episodic care in EDs.

This study defines the extent of ED utilization by PWHDx OOC and confirms the ED as a key setting for identifying and re-engaging individuals otherwise not engaged by conventional care pathways. Among 88,501 adult ED visits across 3 diverse sites, 798 unique PWH were identified—representing 0.9% of all adult encounters. Of these PWH, 5.1% were ND, 21.6% were PWHDx OOC, and 73.3% were PWHDx IC. These data underscore the substantial proportion of ED PWHDx, many of whom remain OOC, highlighting an important opportunity for re-engagement.

Our findings demonstrate that although universal HIV screening remains essential for identifying undiagnosed cases, additional approaches are needed to identify and link PWHDx OOC. Routine screening of 17,710 patients yielded 41 new HIV diagnoses and 168 rescreened PWHDx, whereas an EHRa identified 589 additional PWHDx excluded from screening by design—of whom 113 (19.2%) were OOC. These findings show that an EHR-based strategy can substantially expand the reach of existing ED HIV programs by capturing a large population not detected through screening alone.

At all sites, the screening algorithm was designed to exclude PWHDx from testing; however, of the 209 patients identified through screening, 80.4% were previously diagnosed. These duplicate testing rates are high but consistent with other ED reports.^11^, ^12^, ^13^ Duplicate testing may occur when the algorithm fails to recognize PWHDx because HIV-related laboratory data or PMH fields are incomplete or unavailable. Clinicians may also intentionally order repeat testing to confirm a diagnosis or facilitate re-entry into care.

The EHRa model required no new software or external data exchange and can be implemented in most EDs using EHR platforms with functionality comparable to Epic. By efficiently identifying PWHDx, it reduces reliance on provider-initiated identification, limits repeat testing, and minimizes missed linkage opportunities. This study demonstrates that EHRa is a low-cost, scalable approach adaptable to local workflows and staffing. Many EDs already use automated screening logic, and these systems can generate real-time notifications when a known PWHDx returns to the ED, enabling activation of EHRa workflows to identify and re-engage patients excluded from routine screening. In such a prospective workflow, navigation staff would receive an alert at the time of ED registration, review the patient’s care status using EHRa-generated data, and initiate re-engagement outreach during or immediately following the visit. This study establishes the feasibility and yield of the EHRa as a proof-of-concept; prospective evaluation of real-time implementation, including its effect on linkage rates, ART initiation, and long-term retention in care, represents an essential next step.

Across the 3 sites, demographic and clinical patterns were largely consistent. HIV prevalence among adult ED patients was similar at AHS-Highland (0.9%), AHS-San Leandro (0.7%), and HFH (1.0%). The proportion of PWHDx OOC was lower at the community site (AHS-SLH, 16.8%) than at the academic centers (AHS-HH, 23.0%; HFH, 22.0%), which may reflect differences in patient volume, demographics, or outpatient HIV access. Despite these variations, overall findings were directionally consistent, supporting the generalizability of this model across ED settings.

Previous studies have reported similar success using EHR-based tools to identify PWHDx in EDs.^15^^,^^16^^,^^26^^,^^27^ Ruffner et al. integrated an EHR algorithm with ED HIV/HCV screening to flag patients with prior positive HIV tests or problem-list diagnoses, prompting staff review and follow-up.^15^ Over one year, the program identified 104 PWHDx OOC, 32% of whom were successfully linked to care. Similarly, the University of Chicago implemented a daily EHR-based report across ED and inpatient settings, identifying 393 PWHDx—including 56 (14%) OOC—whereas a concurrent ED screening program identified only 17 ND cases over the same period.^16^ Beyond EHRa, other programs have described automated systems linking ED registries with state surveillance databases to flag OOC patients in real time.^26^^,^^27^ These health information exchange models, although effective and achievable in some jurisdictions, require complex data-sharing agreements and substantial legal compliance, which limit scalability.^25^^,^^28^^,^^29^ In contrast, the EHRa model presented here operates entirely within existing hospital systems, offering a feasible, rapidly deployable alternative.

Compared with prior published approaches, this EHRa has 4 distinguishing features: (1) it functions independently of the HIV screening workflow, targeting PWHDx who are systematically excluded from routine screening; (2) it was designed specifically to identify PWHDx who may be disengaged from care and to quantify the incremental yield of algorithm-based identification beyond routine HIV screening; (3) it was evaluated across multiple EDs within 2 distinct health systems, including both academic and community settings; and (4) it requires no external data exchange or institutional data-sharing agreements, making it more readily scalable than health information exchange–dependent models.

The CD4 and viral load findings underscore the urgency of re-engagement for PWHDx OOC. This group had markedly lower CD4 counts and higher viral loads than those IC, with more than one-third presenting with AIDS and only 17.6% having undetectable viral loads. These results indicate that many ED PWHDx OOC remain viremic and at high risk of transmission, emphasizing the public health importance of identifying and linking them to care.

Rapid ART initiation (rART) in the ED is feasible, safe, and nationally recommended for ND patients.^12^^,^^30^ Same-day ART is associated with >90% uptake, minimal adverse events, and faster viral suppression compared with delayed referral.^12^^,^^31^ Given that nearly half of ED PWHDx OOC are not on ART, expanding ED-based rART to treatment-experienced but disengaged patients warrants further study and may offer important individual and population benefits. Implementing rART for PWHDx OOC will require standardized protocols addressing ART history, resistance risk, and drug interactions, ideally supported by real-time infectious disease consultation.

Our data also highlight the structural and social barriers faced by PWHDx OOC. Compared with those IC, OOC patients were more likely to be unhoused, lack a phone, or be uninsured—each independently associated with disengagement in multivariable analysis. Substance use and mental health disorders were also common, consistent with prior work linking psychosocial instability to poor HIV retention.^26^^,^^32^^,^^33^ Notably, substance use disorder appeared attenuated in the multivariable model despite its high prevalence among OOC patients in bivariate analysis. This attenuation is consistent with the framework that social determinants—particularly housing, insurance coverage, and phone access—function as mediators for retention and engagement. Clinicians and linkage teams increasingly rely on telemedicine and remote outreach, and patients who use drugs are more likely to have difficulty accessing and retaining a cell phone, housing, or insurance coverage. This implies that not only is substance use treatment important for engaging OOC patients, but addressing mediators to accessing care, including provision of cell phones, insurance access, and transportation, is needed to best support this population.

Effective re-engagement will require strategies that extend beyond the ED visit.^5^ Dedicated staff trained in linkage and social-service coordination are critical for addressing these barriers. They can facilitate insurance enrollment, housing referrals, transportation, phone access support, and referrals for behavioral health and substance use treatment, bridging gaps that ED clinicians cannot address within routine workflows.

Importantly, identification of OOC patients in the ED is a necessary but insufficient step. Many patients who are lost to care are not merely logistically disconnected; some are ambivalent or disengaged for complex psychosocial, structural, or experiential reasons, including prior negative encounters with the health care system, stigma, and competing survival priorities. Effective re-engagement in these populations requires patient-centered, culturally responsive approaches that extend well beyond the ED visit and address upstream social determinants directly. The multivariable associations identified in this study—housing instability, lack of phone access, and uninsurance as independent predictors of OOC status—highlight the structural drivers of disengagement and suggest that re-engagement strategies must incorporate housing support, insurance enrollment assistance, and accessible communication pathways to reach the most marginalized individuals.

Routine HIV screening is optimized to reduce unnecessary repeat testing of PWHDx. Without a strategy to identify PWHDx presenting to the ED, many OOC patients will be unrecognized. This multicenter study shows that a simple, locally generated EHRa can identify large numbers of ED PWHDx OOC and support re-engagement efforts. Integrating EHRa tools into ED workflows may represent a feasible and scalable approach to strengthening the HIV care continuum and advancing national EHE goals, although prospective evaluation and local validation will be important before broader implementation. Future work should evaluate real-time implementation, rART initiation, and linkage models that address the social and structural barriers driving care disengagement.

## Author Contributions

DW, BH, MB, MJ, CL, and JM conceived the study. DW, BH, and JM acquired the funding and designed the study. MB, MJ, CR, GR, CM, KD, CL, DB, CL, DB, JR, SP, SZ, WG, IB, and MP collected the data. EA designed the statistical analysis plan and conducted the data analysis. DW drafted the manuscript. BH, JM, IB, JM, EA, and MP contributed substantially to its revision. All authors have read and approved the submitted version.

## Funding and Support

This work was funded by Gilead Sciences’ Frontlines of Communities in the U.S. (FOCUS) program, in part. FOCUS funding supports HIV, HCV, and HBV screening and linkage to a first medical appointment. FOCUS partners do not use FOCUS awards for activities beyond linkage to a first medical appointment. Additional funding to develop this manuscript was provided to BH and DW by Gilead Sciences Inc. with BH and DW retaining final control over the content. The funder played no role in conceptualization, design, data collection, analysis and interpretation of data, or manuscript writing. This manuscript has not been published or presented elsewhere in part or in entirety and is not under consideration by another journal.

## Declaration of Generative AI and AI-Assisted Technologies in the Written Process

During the preparation of this work DW used ChatGPT to assist with word-count reduction, clarity improvements, and final-stage editorial refinement. After using this service, DW reviewed and edited the content as needed and takes full responsibility for the content of the published article.

## Data Sharing Statement

Data supporting the findings of this study are available from the corresponding author upon request.

## Conflict of Interest Statement

DW, JM, and BHJ receive grant funding from Gilead Sciences’ FOCUS Program to support HIV screening and linkage to care activities at their respective sites. Funding from Gilead to BH and DW supported writing only, with authors retaining full editorial control; Gilead had no role in data interpretation or manuscript content decisions. All other authors declare no conflicts of interest.
